# Prevalence of human papillomavirus and HPV vaccination among cisgender bisexual women: Protocol for a systematic review and meta-analysis

**DOI:** 10.1371/journal.pone.0360427

**Published:** 2026-11-10

**Authors:** Kendall A. Lawley, Karthikeya Kusuma, Stephanie A. Farquhar, Steven M. Goodreau, Rachel L. Winer

**Affiliations:** 1 Department of Epidemiology, University of Washington, Seattle, Washington, United States of America; 2 School of Public Health, University of Washington, Seattle, Washington, United States of America; 3 Department of Health Systems and Population Health, University of Washington, Seattle, Washington, United States of America; 4 Department of Anthropology, University of Washington, Seattle, Washington, United States of America; Amrita Vishwa Vidyapeetham - Amritapuri Campus, INDIA

## Abstract

Human papillomavirus (HPV) is the most common sexually transmitted infection in the United States, and a primary cause of cervical cancer. Although cervical cancer is largely preventable through HPV vaccination, vaccine coverage remains suboptimal. Research on HPV prevalence and vaccination among cisgender bisexual women is limited and inconsistent. This may be partly due to variation in how bisexuality is defined. Bisexuality may be operationalized based on sexual identity (e.g., self-identification as bisexual) or sexual behavior (e.g., sexual contact with more than one sex or gender), and these differing definitions may capture distinct populations and yield divergent health findings. This paper proposes a protocol for a systematic review and meta-analysis to examine whether reported HPV prevalence and vaccination rates among cisgender bisexual women differ based on whether bisexuality is defined by identity or behavior. The protocol is registered in PROSPERO (CRD420251252015) and follows PRISMA-P guidelines. Random-effects meta-analyses will estimate pooled HPV prevalence and vaccination rates by definition type. Findings will clarify how operational definitions shape observed HPV-related outcomes and inform tailored cervical cancer prevention strategies for cisgender bisexual women.

## Introduction

Human papillomavirus (HPV) is a DNA virus with more than 200 known types. More than 40 of these infect the epithelial lining of the anogenital tract and are primarily transmitted through sexual contact [1–4]. HPV is the most common sexually transmitted infection in the United States (US). Two high‑risk types, HPV 16 and HPV 18, are responsible for the majority of cervical cancer cases [2]. Cervical cancer is one of the most preventable forms of cancer and yet has the third highest incidence and mortality rate of gynecologic cancers in the US, making it a notable public health concern [5,6].

The primary method of HPV prevention is HPV vaccination [7]. Since its introduction in 2006 the HPV vaccine has demonstrated marked reduction in sexual transmission of HPV infection and subsequent development of cervical cancer [8]. A recent study examining the impact of the HPV vaccine on trends in cervical precancer in the US showed a 79% decrease and an 80% decrease in incidence of cervical intraepithelial neoplasia grades 2 and 3 respectively among 20- to 24-year-olds between 2008 and 2022 [9]. Despite the clear, long-term benefits of the HPV vaccine, HPV vaccine coverage for adolescents in the US remains well below the Healthy People 2030 target of 80% coverage among adolescents ages 13–15, with recent estimates showing 58.7% coverage in 2024 [10].

Currently, bisexuality is thought to be the most prevalent sexual minority identity in the US with adult and adolescent women being the most likely to identify as bisexual [11,12]. As the proportion of women in the US identifying as bisexual continues to increase, the need to understand patterns of HPV infection and vaccination uptake among this population becomes increasingly pressing. Research that specifically focuses on HPV prevalence and HPV vaccination among cisgender bisexual women is scarce, with inconsistent and contradictory findings, making it difficult to draw conclusions about the cervical cancer risk for this population [13–15].

A primary challenge in health research with LGBTQ+ populations, is that studies often aggregate participants with different sexual identities into one broad “sexual minority” category. Similarly, in most research with populations of sexual minority women (SMW), bisexual women are often combined with lesbian women for convenience or greater statistical power. However, this poses a challenge when trying to understand potential health differences between different identity categories. While the studies that differentiate between lesbian women and bisexual women are somewhat limited, there is evidence that there are notable health differences between the two groups. Existing research has demonstrated that SMW’s specific sexual identity is associated with differences in various health outcomes. One study found significant differences in mental health outcomes and hazardous drinking behavior between participants who identified as exclusively lesbian, mostly lesbian, and bisexual [16]. Similarly, a study examining differences in health-related quality of life between lesbian and bisexual women found that bisexual women showed greater mental distress and poorer general health than lesbian women, controlling for sociodemographic characteristics, health care access, and health behaviors [17]. There is a need for more studies that consider lesbian women and bisexual women as two distinct populations due to considerable observed differences between the two groups. Based on the findings from previous studies, it stands to reason that examining HPV prevalence and vaccination for bisexual women specifically separate of lesbian women may be warranted. Bisexual women and lesbian women are likely to have different patterns of sexual behavior and sexual partnerships, and as a result, are likely to have different risks, or perceived risks, related to HPV and cervical cancer. Similarly, there is research to suggest that SMW have low engagement in HPV screening and vaccination, particularly in comparison to heterosexual women [18,19]. However, it is unclear if disparities in engagement are consistent across sexual minority identity categories or if certain identities are primarily driving these disparities [13–15,20].

One potential reason why there may be inconsistencies in findings related to HPV and cervical cancer among bisexual women could be that this population is inherently challenging to define [13–15,21,22] Bisexuality is difficult to operationalize and there is not one consistent definition used in LGBTQ+ health literature [12].

Definitions of bisexuality can typically be divided into two categories: definitions related to sexual *behavior* and definitions related to sexual *identity* [12] Behavioral definitions of bisexuality consider the sex or gender of someone’s sexual partners. One example of this could be, defining any person who has ever had sexual contact with people of more than one sex or gender as bisexual regardless of how they conceptualize their own sexual orientation. Identity-based definitions consider someone’s perception of their own attraction or identity, regardless of whether they have had sexual partners of more than one sex or gender. Different definitions of bisexuality can capture results from different people and can draw different conclusions around certain health conditions. While these definitions overlap, each definition on its own would exclude participants who could provide meaningful results for a research study on bisexuality and sexual health.

Some previous works estimate that over 80% of bisexual individuals primarily or exclusively engage in opposite-sex romantic and sexual partnerships despite reporting attraction to more than one sex or gender [23]. Because individuals who identify as bisexual may not necessarily have sexual partners of more than one sex or gender, there may be greater inconsistency in HPV-related outcomes among bisexual women when using an identity-based definition of bisexuality [24]. It is possible that there are meaningful differences in reported HPV prevalence and HPV vaccination uptake between bisexual-identifying women with sexual partners of different sexes or genders compared to bisexual-identifying women with sexual partners of predominantly one sex or gender, and these women are likely to have unique needs related to HPV prevention and care that differ from the needs of their counterparts who identify as lesbian and heterosexual.

The current protocol is for a proposed systematic review and meta-analysis that aims to examine whether there are differences in reported HPV vaccination and HPV prevalence among cisgender bisexual women in research studies based on how the authors operationally define bisexuality (identity vs behavior). We hypothesize that studies that define bisexuality based on reported sexual behavior will report higher HPV prevalence and higher HPV vaccination than studies that define bisexuality based on participants’ self-reported sexual orientation.

## Methods

### Study registration

This protocol was registered in the International Prospective Register of Systematic Reviews database (PROSPERO) on February 2, 2026 (CRD420251252015) [25] and was developed using guidelines of Preferred Reporting Items for Systematic Review and Meta-Analyses Protocols (PRISMA-P) found in S1 Table [26].. Any potential amendments made to the protocol will be documented and uploaded to PROSPERO and discussed in the final manuscript for full transparency of the study process.

### Search strategy

Searches will be run using Embase, PubMed, and Google Scholar to identify relevant papers. Additionally, the reference lists of any papers that are included in the final review will be manually searched for additional publications that may have been overlooked during the initial review process. Embase and PubMed were selected as they are commonly used in systematic reviews related to public health and biomedical research. Google Scholar was selected as an additional database due to its ability to identify large amounts of grey literature such as white papers, government reports, and doctoral dissertations [27]. Grey literature is advantageous to include in many systematic reviews as it can reduce publication bias, and can be especially beneficial for systematic reviews such as this one, that focus on niche research subjects [28]. Additionally, Embase, PubMed, and Google Scholar together have been found to have a high level of search precision for systematic reviews, while balancing the number of articles that researchers conducting the review must read [29]. A research librarian was consulted to identify the most appropriate key words to identify articles of interest. The exact keywords used as search terms can be found in Table 1. Papers that are identified through the database searches will be uploaded to Covidence, a program designed to manage articles for systematic reviews. All potential abstracts will be uploaded to Covidence where duplicate abstracts will be automatically removed by the Covidence system. Uploaded abstracts will be assessed for relevance and abstracts that are deemed potentially relevant will receive a full text review. Two separate investigators (KAL and KK) will review potential articles for relevance. The reviewers will examine the abstracts that are uploaded to Covidence to assess relevance and inclusion. Each reviewer will then assess the full texts for relevance. Any discrepancies will be discussed between the reviewers until consensus is reached. After all articles have been decided upon, the reviewers will perform the extraction of required variables from each study to be included. Multiple variables will be used for data abstraction. Table 2 provides the variables and variable definitions that will be used to guide data abstraction.

**Table 1:** Search terms to be used in the meta-analysis.

Search terms
((“human papillomavirus viruses”[MeSH Terms] OR “human papillomavirus”[tw] OR hpv[tw] OR “Papillomavirus Infections”[Mesh]) AND (“Vaccination”[Mesh] OR “Vaccination”[tw] OR “Vaccines”[Mesh] OR Vaccine*[tw])OR “Papillomavirus Vaccines”[Mesh]) AND (bisexual wom*[tw] OR ((“Bisexuality”[Mesh] OR sexual minority[tw] OR “sexual and gender minorities”[MeSH Terms]) AND (“women”[MeSH Terms] OR (female[tw] OR “female”[MeSH Terms]))))

**Table 2:** Variables used for data abstraction in the review process.

Variables	Definition
Year	Publication year will be identified and extracted. There will be no specific year cutoff. Studies from any year are eligible to be included.
Geographic Region	Studies will not be limited by country but will be limited to papers that are written in English. Geographic regions will be extracted to examine potential differences by geography. It is possible that countries with socialized health care may have different outcomes than countries without. It is unlikely that there will be enough countries represented in this review to make quantitative comparisons by geography, but after point estimates have been acquired for each study, qualitative comparisons will be examined between countries with versus without universal healthcare using a WHO global index of universal health care.
Sample Size	Sample size will be extracted from each study. Studies with larger sample sizes will be given more weight in the analyses than studies with smaller sample sizes. Sample sizes will also be necessary for determining the denominators in each study which will be used to calculate prevalence if the exact prevalence is not already provided in the study. Because the number of papers on this topic is likely limited, there will not be a minimum sample size for inclusion in the meta-analysis.
Recruitment/Sampling Strategy	Study recruitment and sampling strategy will be extracted to identify potential bias that may be introduced based on a study’s participant selection. Studies with random sampling will have less sampling bias than studies with convenience sampling. This will be recorded to include random effects in the meta-analytic model and to conduct a quality assessment of included studies using Cochrane’s risk of bias assessment protocol.
Age Range	Participant age range will be extracted from each study. The sample age may reflect differences in HPV vaccination recommendations and protocols as the recommendations for who should get vaccinated and when vaccination should occur have changed over time.
Race/Ethnicity	When available, participant race/ethnicity will be recorded and if there is sufficient information, will be included as a covariate in the model.
HPV Prevalence	HPV prevalence will be extracted and used as an outcome variable.
HPV Type	When available, information on HPV types reported in the studies will be recorded. Testing for certain HPV types can influence reports of HPV prevalence and this will be considered and examined for potential importance.
HPV Assay	When available, information on HPV assays used in the studies will be recorded. HPV test assays can similarly influence reports of HPV prevalence and this will be considered and examined for potential importance.
HPV Vaccination	HPV vaccination will be extracted and used as an outcome variable. Participants who received any number of doses of the HPV vaccine will be considered vaccinated even if they did not complete the vaccine series.
Definition of Bisexuality	The way a study defines bisexuality (identify vs behavior) will be extracted and used as the primary predictor of HPV prevalence and HPV vaccination.

### Inclusion and exclusion criteria

In order to be included in the proposed systematic review, studies must meet the following criteria: written in English, include information regarding rates of HPV vaccination and/or prevalence of HPV infection, use a sample that includes cisgender women who either identify as bisexual or who are classified as behaviorally bisexual in the study, and specify the form(s) of operationalization for the definition of bisexuality.

### Data extraction

All data extraction activities will be conducted using Covidence [30]. Covidence is an online platform designed to streamline and support the systematic review process. Papers that are identified through the literature search will be uploaded to Covidence to manage search results and determine each paper’s review eligibility. Once articles have been identified and uploaded to Covidence, abstracts will be reviewed and assessed for relevance. Articles that are deemed relevant through abstract review will receive a full text review. Articles that are selected from the full text review will have data extracted and effect sizes calculated.

### Data analysis and synthesis

Two outcomes of interest will be evaluated: reported prevalence of HPV infection and HPV vaccination. Once data are extracted from included studies, a meta-analysis will be conducted to calculate the overall point estimates of HPV prevalence and HPV vaccination for papers that report bisexual identity and the overall estimate of HPV prevalence and vaccination for papers that report bisexual behavior. If a study provides an identity-based definition as well as a behavior-based definition, that study will be categorized as using a behavior-based definition. This is because behavior-based definitions provide more information regarding HPV risk than identity-based definitions, as they specifically state that a participant has had sexual contact with people of more than one sex or gender. Identity-based definitions provide no additional information about sexual history. A multi-level random effects model will be used to account for any potential heterogeneity and interdependency of effect sizes between studies [31] Multilevel meta-analytic techniques enable the inclusion of all effect sizes by statistically accounting for hierarchical data where effect sizes are nested within studies [32]. This is done by modeling three difference sources of variance for each study: sampling variance for the observed effect sizes, variance between effect sizes calculated from the same dataset, and variance between studies.

Prevalence will be calculated for each study and weighted by study sample size. Studies will then be pooled to obtain overall point estimates for each of the two groups independently, meaning there will be the pooled prevalence of HPV diagnosis and vaccination for studies that define bisexuality based on identity as well as the pooled prevalence of HPV diagnosis and vaccination for studies that define bisexuality based on behavior. Point estimates and 95% confidence intervals between the two groups will be compared and a single pooled point estimate that combines both definitions of bisexuality will be used in a meta-regression to statistically assess whether the operational definition of bisexuality is associated with HPV prevalence and HPV vaccination [33] I^2^ statistics and prediction intervals will be calculated to assess statistical heterogeneity, and funnel plots and Egger’s test will be utilized to assess risk of publication bias among the included studies [34–36]. Depending on the number of studies included in the final analyses and the specificity of information provided in these studies, subgroup analyses may be conducted. Subgroup analyses of particular interest include differences across geographic regions, race/ethnicity, and age range. All analyses will be conducted using R and stored in a GitHub repository that will be made available once the meta-analysis has been published. If the review does not yield enough studies to reasonably conduct meaningful quantitative analyses, the results will be qualitatively summarized and described to provide preliminary conclusions on the synthesized evidence.

### Risk of bias assessment

Quality assessment will be performed using the Risk of Bias in Non-Randomized Studies of Exposures (ROBINS-E) tool, a validated risk of bias tool created by Cochrane Review [37] The ROBINS-E assessment was designed to assess the risk of bias in observational epidemiologic studies that are included in systematic reviews. This tool assesses potential bias in seven different domains (confounding, measurement of the exposure, participant selection, post-exposure interventions, missing data, outcome measurement, reporting of results), which are assessed in order to evaluate the overall risk of bias in included studies. The ROBINS-E tool was selected for the current study due to its flexibility and its application to multiple study designs. Given the focus of the proposed systematic review, it is necessary to use a risk of bias assessment tool that can accommodate multiple types of observational study designs. However, other risk of bias assessments that are designed for prevalence-based meta-analyses may not be entirely appropriate in this context. While the current study aims to examine the prevalence of the outcomes of interest (HPV and HPV vaccination), it will also be comparing the prevalence between two separate “exposure” groups; specifically, participants defined as bisexual based on identity versus participants defined as bisexual based on behavior. It is especially important in this context to utilize a risk of bias tool that assesses for bias in a study’s exposure, because the primary hypothesis of the proposed systematic review is that reported prevalence of HPV infection and HPV vaccination may differ based on what information is being used to classify a woman as bisexual. Additionally, the strength of the body of evidence included in the review will be assessed using the Grading of Recommendations Assessment, Development, and Evaluation (GRADE) tool [38]. Following the same structure outlined in the search strategy, two separate investigators (KAL and KK) will perform Risk of Bias assessments on all included studies. Any discrepancies will be discussed between the reviewers until consensus is reached. If consensus is unable to be reached, the two reviewers will request input from the rest of the team.

## Proposed timeline

At the present time, record screening, data extraction and writing of the results have not begun. The proposed timeline for each element of the systematic review process can be found in Table 3.

**Table 3:** Proposed timeline for each step of the systematic review.

Study Stage	Estimated Start Date	Estimated End Date
Record Screening	June 2026	August 2026
Data Extraction	August 2026	November 2026
Risk of Bias Assessment	November 2026	December 2026
Data Synthesis	December 2026	February 2027

## Discussion

The proposed systematic review and meta-analysis will be conducted to examine whether the prevalence of HPV infection and HPV vaccination differ among cisgender bisexual women based on how bisexuality is defined in each included study. Given the importance of reducing the spread of HPV in the prevention of cervical cancer and the paucity of information on HPV vaccination in the population of interest, this study aims to explore how bisexual behavior and bisexual identity may each be independently related to HPV infection and vaccination. The review will provide foundational information that can inform subsequent HPV intervention strategies tailored to the specific needs of cisgender bisexual women.

## Supporting information

S1 Table Adapted preferred reporting items for systematic review and meta-analysis protocols checklist. (DOCX)
